# Supplementary material for: Optimized Method for the Identification of Candidate Genes and Molecular Maker Development Related to Drought Tolerance in Oil Palm (Elaeis guineensis Jacq.)
Source: Plants (Basel). 2022 Sep 4;11(17):2317. doi: 10.3390/plants11172317 (PMC9460821; doi:10.3390/plants11172317)
Supplement: Supplementary file 1 [file plants-11-02317-s001.zip › plants-1897770-supplementary.pdf]

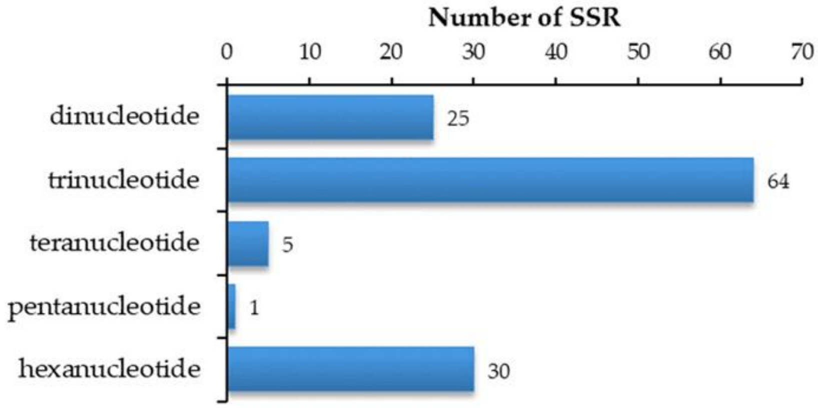

Supplementary Figure S1. The distribution of repeated sequence.

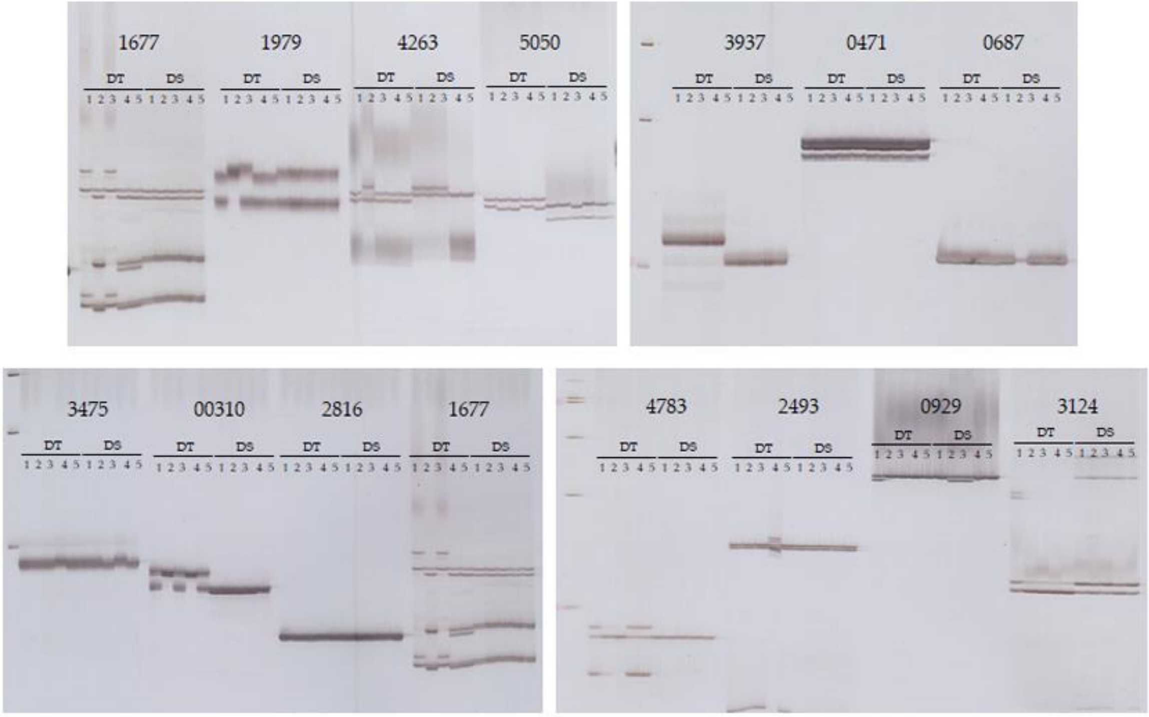

Supplementary Figure S2. Polymorphism banding of five primers that clearly distinguished the differentiation between drought tolerant and drought-sensitive variety when separated on 5% denaturing polyacrylamide gel electrophoresis.

Supplementary Table S1. Nucleotide sequence obtain form PCR product of leaf and root of oil palm dehydrated for 90 days compared with NCBI database via BASTN

DNA banding name	Size (bp)	Similarity	Accession no.	E-value	species	% similarity
O2A2-L4	324	PREDICTED: *Phoenix dactylifera* histone H2A (LOC103696590), mRNA	XM_008778268.1	3e-114	*Phoenix dactylifera*	89% (289/325)
O2A2-L9	258	PREDICTED: *Phoenix dactylifera* protein FEZ-like (OC103703150), mRNA	XM_008785901.1	2e-83	*P. dactylifera*	95% 193/204)
O2A2-L11	190	PREDICTED: *Phoenix dactylifera* transcription factor bHLH106-like (LOC103705042), mRNA	XM_008788632.1	1e-52	*P. dactylifera*	97% 125/129)
O2A2-R4	290	*Elaeis guineensis* cysteine proteinase (CPRS1) mRNA, complete cds	EF622021.1	4e-104	*E. guineensis* Jacq	99% 217/219)
O2A2-R8	226	PREDICTED: *Phoenix dactylifera* putative germin-like protein 2-1 (LOC103719636), mRNA	XM_008808973.1	9e-36	*P. dactylifera*	91% 104/114)
O2A2-R9	220	PREDICTED: *Phoenix dactylifera* uncharacterized LOC103716595 (LOC103716595), transcript variant X1, ncRNA	XR_605548.1	3e-42	*P. dactylifera*	79% (173/219)
O2A5-L3	417	*Chroococcidiopsis thermalis* PCC 7203, complete genome	CP003597.1	6e-130	*Chroococcidiopsis thermalis*	85% 354/415)
O2A5-L9	275	*Phoenix dactylifera* mitochondrion, complete genome	JN375330.1	1e-135	*P. dactylifera*	99% (274-275)
O2A5-L11	264	*PREDICTED*: Citrus sinensis uncharacterized LOC102626228 (LOC102626228), mRNA	XM_006464828.1	1e-27	*Citrus sinensis*	76% 139/183)
O2A5-L17	201	*Oryza granulate clone OG_ABa0049D11, complete sequence*	AC240779.1	1e-14	*Oryza granulata*	74% (102/137)
O2A5-R4	239	PREDICTED: *Phoenix dactylifera* primary amine oxidase-like (LOC103706132), mRNA	XM_008790138.1	4e-09	*P. dactylifera*	74% (81/109)
O4A3-L1	399	*PREDICTED*: *Phoenix dactylifera* plasminogen activator 1RNA-binding protein-like (LOC103721814), mRNA	XM_008812160.1	1e-125	*P. dactylifera*	88% (325/368)
O4A3-L2	236	*PREDICTED: Phoenix dactylifera 3'-N-debenzol-2'-deoxytaxol N-benzoyltransferase-like* (LOC103720896), *mRNA*	XM_008810860.1	5e-27	*P. dactylifera*	82% 110/1340
O4A3-L4	649	*Elaeis guaineensis disrupted DEF1 gene, partial sequence; and retrotransposons RLG_Koala_Eg133H20-1 and RLC_Rider_Eg133H20-1, complete sequence*	KF142646.1	7e-138	*E. guineensis Jacq*	84% (387/460)

Supplementary Table S1. Nucleotide sequence obtain form PCR product of leaf and root of oil palm dehydrated for90 days compared with NCBI database via BASTN. (cont.)

DNA banding name	Size (bp)	similarity	Accession no.	E-value	species	% similarity
O4A3-L6	521	*Theobroma cacao* PIF1 helicase, putative (TCM016903) mRNA, complete cds	XM_007031435.1	1e-15	*Theobroma cacao*	72% (126/175)
O4A3-L12	382	PREDICTED: *Phoenix dactylifera* transcription initiation factor TFIID subunit 8-like (LOC103695623), mRNA	XM_008776988.1	1e-04	*P. dactylifera*	92% 936/39)
O4A3-L13	305	*Phoenix dactylifera* ATP sulfurylase 1, chloroplastic-like (LOC103720197), transcript variant X2, mRNA	XM_008809802.1	4e-36	*P. dactylifera*	74% 9201/27)
O4A3-L14	351	*Elaeis guaineensis* repetitive DNA, clone pEgkB17	AJ271979.1	3e-82	*Elaeis guaineensis* Jacq	85% 241/283)
O4A3-L16	311	PREDICTED: *Phoenix dactylifera* ATP sulfurylase 1, chloroplastic-like (LOC103720197), transcript variant X2, mRNA	XM_008809802.1	8e-32	*P. dactylifera*	73% 198/273)
O4A3-L17	330	*Populus trichocarpa* clone POP081-J22, complete sequence	AC216474.1	8e-07	*Populus trichocarpa*	85% (47/55)
O4A3-L20	353	PREDICTED: *Phoenix dactylifera* peroxisome biogenesis protein 3-1-like (LOC103723476), transcript variant X2, mRNA	XM_008814395.1	7e-46	*P. dactylifera*	91% 130/143)
O4A3-L25	200	PREDICTED: *Phoenix dactylifera* alpha, alpha-trehalose-phosphate synthase [UDP-forming] 6-like (LOC103706802), transcript variant X5, mRNA	XM_008791043.1	2e-74	*P. dactylifera*	94% 178/189)
O4A3-L33	248	PREDICTED: *Phoenix dactylifera* probable protein phosphatase 2C 23 (LOC103696816), mRNA	XM_008778537.1	4e-22	*P. dactylifera*	95% (69/73)
O4A3-L39	225	PREDICTED: *Phoenix dactylifera* probable LRR receptor-like serine/ threonine-protein kinase At3g47570 (LOC103724159), mRNA	XM_008815330.1	3e-74	*P. dactylifera*	91% 186/204)
O4A3-L50	186	*Elaeis guaineensis* disrupted DEF1 gene, partial sequence; and retrotransposons RLG_Koala_Eg133H20-1 and RLC_Rider_Eg133H20-1, complete sequence	KF142646.1	3e-28	*E. guaineensis* Jacq	78% 143/184)

Supplementalry Table S2. Nucleotide sequence obtain form PCR product of leaf and root of oil palm dehydrated for 90 days compared with NCBI database via BASTX.

DNA banding name	Function	size (bp)	Sequence similarity	BLASTX				
				Accession no.	E-value	species	Amino acid	% similarity
O2A2-L4	Histone acetylation and deacetylation	324	histone H2A	XM_010907423.1	1e-05	*E. guineensis*	22	95% (21/220)
O2A2-L9	Cell division	258	PREDICTED: protein FEZ-like	XP_008784123.1	1e-38	*P. dactylifera*	67	99% (67/68)
O2A2--L11	Transcription process	190	PREDICTED: transcription factor bHLH106-like	XP_008786854.1	2e-18	*P. dactylifera*	42	95% (42/44)
O2A2-R4	Metabolic processes	290	cysteine proteinase	ABR19829.1	0.001	*E. guineensis*	15	100% (15/15)
O2A5-L3	Adenylate cyclase activity	417	Putative adenylate/guanylate cyclase	WP_015152518.1	5e-77	*C. thermalis*	118	86% (118/138)
O2A5-L9	-	275	Hypothetical protein MTR 1g006860	XP_003588402.1	9e-09	*Medicago truncula ta*	25	83% (25/30)
O2A5-L11	DNA integration	264	Retrotransposon protein, putative unclassified	ABF96015.1	8e-32	*O. sativa* Japonica group	53	62% (53/85)
O2A5-L17	-	201	PREDICTED: uncharacterized protein LOC102607673	XP_006486503.1	5e-19	*C. sinensis*	41	65% (41/63)
O4A3-L2	Transferase activity	236	3'-N-debenzoyl-2'-deoxytaxol N-benzoyltransferase	XP_008778035.1	3e-06	*P. dactylifera*	18	62% (18/29)
O4A3-L4	-	649	Hypothitical protein VITISV 040104	CAN76612.1	4e-18	*V. vinifera*	45	44% (45/103)
O4A3-L6	DNA helicase	521	PIF1 helicase, putative	XP_007031497.1	6e-37	*T. cacao*	75	49% (75/153)
O4A3-L13	-	305	PREDICTED: uncharacterized protein LOC103714969	XP_008800680.1	3e-27	*P. dactylifera*	54	58% (54/93)
O4A3-L16	-	311	PREDICTED: uncharacterized protein LOC103714969	XP_008800680.1	2e-28	*P. dactylifera*	56	57% (56/98)
O4A3-L19	Protein RNA-binding activity	323	PREDICTED: pentatricopeptide repeat-containing protein At4g22760	XP_008803996.1	2e-06	*P.dactylifera*	21	45% (21/47)

Supplementary Table S2. Nucleotide sequence obtain form PCR product of leaf and root of oil palm dehydrated for 90 days compared with NCBI database via BASTX. (cont.)

DNA banding name	Function	size (bp)	Sequence similarity	BLASTX				
				Accession no.	E-value	species	Amino acid	% similarity
O4A3-L20	Peroxisome	353	PREDICTED: peroxisome biogenesis protein 3-2-like	XP_008803262.1	3e-13	*P. dactylifera*	35	73% (35/480)
O4A3-L25	Trehalose synthesis	200	PREDICTED: alpha, alpha-trehalose-phosphate synthase [UDP-forming] 6-like isoform X1	XP_008789260.1	2e-14	*P. dactylifera*	38	86% (38/44)
O4A3-L38	Signaling	226	Serine/threonine-protein phosphatase 7 long form homolog	XP_004496822.1	8e-06	*Cicer arietinum*	19	44% (19/43)
O4A3-L39	Signaling	225	PREDICTED: probable LRR receptor-like serine/threonine-protein kinase At3g47570	XP_008813552.1	2e-20	*P. dactylifera*	59	88% (59/67)
O4A3-L41	-	221	PREDICTED: uncharacterized protein LOC103697118	XP_008777135.1	8e-07	*P. dactylifera*	27	42% (27/64)

Supplementary Table S3. BLASTX from pyrosequencing technology of oil palm after water deficit for 45 days.

No	Protein	Copy No.	
		DT-01	ST-02
1	Putative senesce-associated protein	20	5
2	Transcription factor, putative	78	12
3	Putative MYB transcription factor	2	3
4	MYBR domain transcription factor	1	-
5	Putative R2R3-MYB transcription factor	1	-
6	Transcription factor R2R3-MYB1	3	-
7	Transcription factor R2R3-MYB2	-	1
8	Transcription factor MYB1	-	3
9	Transcription factor 1R-MYB1	1	1
10	ANAC083 transcription factor	4	3
11	NAC domain transcription factor	1	-
12	bZIP family transcription factor	7	2
13	Transcription factor AP2D9	2	1
14	MADS-box transcription factor	7	-
15	Type 1 MADS-box transcription factor	6	-
16	AP2/ERF domain-containing transcription factor	2	2
17	AP2/EREBP transcription factor, putative	1	-
18	Heat stress transcription factor A-1	4	1
19	Heat stress transcription factor A-2c	1	-
20	Heat stress transcription factor A3	1	-
21	Ethylene transcription factor	1	-
22	WRKY transcription factor, putative	1	2
23	WRKY transcription factor 7	-	1
24	WRKY1 transcription factor 20	-	5
25	WRKY1 transcription factor 21	1	-
26	WRKY transcription factor 24	-	2
27	WRKY transcription factor 27	1	-
28	WRKY domain transcription factor	2	-
29	Transcription factor WRKY-1	3	-
30	WRKY51-superfamily of TFs having WRKY and zinc finger domain	2	-
31	Putative alfin-like transcription factor	1	3
32	BTF3b-like transcription factor	2	1
33	bHLH transcription factor	8	9
34	Transcription factor jumonji domain-containing protein	1	-
35	HD domain class transcription factor	2	2
36	GRAS family transcription factor	5	2
37	TLP domain class transcription factor	3	-
38	CAMTA domain class transcription factor	2	-
39	SCL domain class transcription factor	3	-
40	Betain aldehyde dehydrogenase	2	-
41	Pyrroline-5-carboxylate reductase	1	3

Supplementary Table S3. BLASTX from pyrosequencing technology of oil palm after water deficit for 45 days. (cont.)

No	Protein	Copy No.	
		DT-01	ST-02
42	1--pyrroline-5-carboxylate dehydrogenase, putative	1	-
43	Delta-1- pyrroline-5-carboxylate dehydrogenase	5	-
44	Trehalose-6-phosphate synthase	6	11
45	Glucose-6-phosphate isomerase	2	4
46	Mannitol 2-dehydrogenase [$NADP^+$]	2	-
47	Superoxide dismutase	3	1
48	Copper/Zinc Superoxide dismutase	5	1
49	Fe-superoxide dismutase	1	3
50	Copper chaperone for superoxide dismutase	1	1
51	Catalase	2	6
52	Catalase 2	1	1
53	Ascorbate peroxidase	2	-
54	Monodehydroascorbate reductase	3	2
55	Peroxisome type ascorbate peroxidase	1	4
56	Cytosolic ascorbate peroxidase	3	3
57	Cytosolic ascorbate peroxidase 2	2	-
58	Nucleobase ascorbate transportor	4	-
59	Peroxidase	3	2
60	Peroxidase 65 precursor, putative	-	1
61	Peroxidase 73 precursor, putative	-	1
62	Thiorioxin peroxidase	1	1
63	Glutathione reductase, putative	5	7
64	Glutathione transferase, putative	2	2
65	Glutathione transferase III(b)	3	2
66	Glutathione S-transferase	3	9
67	Glutathione S-transferase Cla47	1	-
68	Glutathione peroxidase	4	5
69	Phospholipid hydroperoxidase glutathione peroxidase	1	-
70	Tau glutathione S-transferase	3	-
71	Chloroplastic glutathione reductase	1	-
78	Protein phosphatase 2C	10	4
79	Protein phosphatase 2C containing protein	1	2
80	NAC-like protein	3	3
81	NAC domain protein	4	5
82	NAC protein 1	2	3
83	MYB	1	-
84	MYB 1	-	3
85	MYB 6	-	3
86	MYC2 bHLH protein	3	-
87	Helicase, putative	6	2
88	Helicase domain-containing protein	7	-

Supplementary Table S3. BLASTX from pyrosequencing technology of oil palm after water deficit for 45 days. (cont.)

No	Protein	Copy No.	
		DT-01	ST-02
89	ATP-dependent RNA helicase	11	4
90	DEAD-box ATP-dependent RNA helicase, putative	17	14
91	DEAD-box RNA helicase	3	-
92	Chromodomain helicase DNA binding protein	4	-
93	RNA helicase-like protein	3	1
94	Ethylene response factor	3	4
95	Ethylene response protein	1	-
96	Ethylene response 3	4	-
97	Ethylene responsive transcription factor 2a	-	1
98	Ethylene responsive transcriptional coactivator	5	-
99	Putative ethylene-responsive elongation factor EF-Ts precursor	1	1
100	Ethylene receptor	1	5
101	Ethylene response sensor 1	2	-
102	Beta-galactosidase	7	8
103	Beta-D-galactosidase	5	4
104	Alkaline alpha galactosidase	1	-
105	Oil palm polygalacturonase allergen PEST 459	10	-
106	Polygalacturonase 2	1	-
107	Polygalacturonase-1 non-catalytic subunit beta precursor, putative	1	-
108	Heat shock protein, putative	4	4
109	Heat shock binding protein	8	-
110	Heat shock protein 101	1	1
111	101 kDa Heat shock protein	2	-
112	Heat shock protein 90	2	1
113	Heat shock protein 70	3	5
114	Heat shock protein 70 (HSP70)-interacting protein, putative	3	8
115	Heat shock protein DnaJ	2	-
116	Heat shock protein DnaJ, N-terminal	3	1
117	Small heat shock protein, chloroplastic	3	4

Supplementary Table S4. Gene ontology analysis of repeated base sequence at GO level 2 of oil palm under water deficit for 45 days.

GO-IDs	GO-term	Seqs
**Biological process**		
GO:0009987	cellular process	49
GO:0008152	metabolic process	49
GO:0044699	single-organism process	25
GO:0065007	biological regulation	13
GO:0071840	cellular component organization or biogenesis	12
GO:0050789	regulation of biological process	12
GO:0050896	response to stimulus	11
GO:0051179	localization	8
GO:0032502	developmental process	5
GO:0032501	multicellular organismal process	5
GO:0023052	signaling	3
GO:0022414	reproductive process	3
GO:0000003	reproduction	3
GO:0048519	negative regulation of biological process	2
GO:0098754	detoxification	1
**Molecular function**		
GO:0005488	binding	32
GO:0003824	catalytic activity	27
GO:0005215	transporter activity	4
GO:0005198	structural molecule activity	4
GO:0001071	nucleic acid binding transcription factor activity	2
GO:0009055	electron carrier activity	2
GO:0000988	transcription factor activity, protein binding	1
GO:0016209	antioxidant activity	1
**Cellular component**		
GO:0044464	cell part	55
GO:0005623	cell	55
GO:0043226	organelle	42
GO:0044422	organelle part	19
GO:0016020	membrane	18
GO:0032991	macromolecular complex	15
GO:0044425	membrane part	13
GO:0031974	membrane-enclosed lumen	2
GO:0009295	nucleoid	2
GO:0099080	supramolecular complex	1
GO:0030054	cell junction	1
GO:0055044	symplast	1

Supplementary Table S5. Comparison of expressed gene associated with drought tolerance from DDRT-RT and pyrosequencing database.

Seq. name	Seq. Size (bp)	DS				DT				Blastx with Predicted CDS from MPBO	NCBI Accession No.	Blastx with NCBI	Pyrosequencing
		Isotig name	Isotiq length	E-value	% homology	Isotig name	Isotiq Size	E-value	% homology			DDRT	
O2A2-L4	324	isotig00907	576	e-146	99%	-	-	-	-	p5.00_sc00270_p0015	XM_010907423.1	histone H2A	histone H2A
O2A2-L11	190	-	-	-	-	-	-	-	-	p5.00_sc00189_p0010	XP_010931526.1	transcription factor bHLH106	-
O2A2-R4	290	isotig01193	1438	0.00	100%	isotig01724	1440	0.00	100%	p5.00_sc00100_p0051	ABR19829.1	cysteine proteinase	oryzain gamma chain-like /cysteine proteinase
O4A3-L6	521	-	-	-	-	-	-	-	-	p5.00_sc00018_p0147	XP_020080716.1	ATP-dependent DNA helicase PIF1-like	-
O4A3-L19	323	isotig03948	393	3.00e-35	55%	isotig04518	598	2.00e-16	32%	P 5.00_sc00009_p0130	XP_039139688.1	pentatricopeptide repeat-containing protein At4g22760-like	pentatricopeptide repeat-containing protein At1g04840
O4A3-L25	200	isotig00740	949	4.00e-99	87.5%	-	-	-	-	p5.00_sc00004_p0342	XP_010932535.1	alpha, alpha-trehalose-phosphate synthase [UDP-forming] 6	trehalose-6-phosphate synthase, putative
O4A3-L38	226	-	-	-	-	isotig03061	821	1.00e-117	92%	p5.00_sc00001_p0208	XP_004496822.1	Serine/threonine-protein phosphatase 7 long form homolog	Serine/threonine protein phosphatase PP1, putative, expressed
O4A3-L39	225	-	-	-	-	-	-	-	-	p5.00_sc00001_p0001	XP_008813552.1	probable LRR receptor-like serine/threonine-protein kinase At3g47570	-

Supplementary Table S6. Allele and genotype frequency of drought-tolerance and drought-sensitive separated by difference primers.

Primers	Varieties	Allele frequency				Genotype frequency									
		A	B	C	D	*AA*	*AB*	*AC*	*AD*	*BB*	*BC*	*BD*	*CC*	*CD*	*DD*
isotig03010	Lamé	0.7	0.3	·	·	0.4	0.6	·	·	·	·	·	·	·	·
	Surathani 1	·	1.0	·	·	·	·	·	·	1.0	·	·	·	·	·
	all of alleles	0.7	1.3	·	·	0.4	0.6	·	·	1.0	·	·	·	·	·
isotig03937	Lamé	1.0	·	·	·	1.0	·	·	·	·	·	·	·	·	·
	Surathani 1	·	1.0	·	·	·	·	·	·	1.0	·	·	·	·	·
	all of alleles	1.0	1.0	·	·	1.0	·	·	·	1.0	·	·	·	·	·
isotig04263	Lamé	0.1	0.4	0.5	·	·	·	0.2	·	·	0.8	·	·	·	·
	Surathani 1	0.3	0.7	·	·	·	0.6	·	·	0.4	·	·	·	·	·
	all of alleles	0.4	1.1	0.5	·	·	0.6	0.2	·	0.4	0.8	·	·	·	·
isotig04783	Lamé	0.3	0.7	.	.	.	0.6	.	.	0.4	.	.	.	.	.
	Surathani 1	.	1.0	.	.	.	.	.	.	1.0	.	.	.	.	.
	all of alleles	0.3	1.7	.	.	.	0.6	.	.	1.4	.	.	.	.	.
isotig05050	Lamé	0.5	0.2	0.3	.	.	0.4	0.6	.	.	.	.	.	.	.
	Surathani 1	.	0.4	0.2	0.4	.	.	.	.	.	0.2	0.6	.	0.2	.
	all of alleles	0.5	0.6	0.5	0.4	.	0.4	0.6	.	.	0.2	0.6	.	0.2	.

Supplementary Table S7 The alignment of polymorphic EST-SSR primer with oil palm reference genome.

Isotig Number	BLASTX			
	**Gene Description**	**NCBI Sequence ID**	**E-value**	**MPOB**
Isotig03101	DEAD-box ATP-dependent RNA helicase 46 [*Elaeis guineensis*]	XP_010941500.1	9e-70	p5.00_sc00010_p0030
Isotig03937	spliceosome-associated protein 130 A [*Elaeis guineensis*]	XP_010939835.1	1e-65	p5.00_sc00036_p0020
Isotig04263	uncharacterized protein LOC105040638 [*Elaeis guineensis*]	XP_010915560.1	4e-57	p5.00_sc00001_p0241
Isotig04783	TATA-binding protein-associated factor 2N isoform X1 [*Elaeis guineensis*]	XP_010925244.1	3e-61	p5.00_sc00039_p0021
Isotig05050	polyadenylate-binding protein-interacting protein 7 [*Elaeis guineensis*]	XP_010917460.1	2e-36	p5.00_sc00064_p0097

Supplementary Table S8 Oligo (dT) primers and arbitrary primers sequencing used for DDRT-PCR.

Primers	Sequence
oligo (dT) 1	5′-CATTATGCTGAGTGATATCTTTTTTTTTAC-3′
oligo (dT) 2	5′-CATTATGCTGAGTGATATCTTTTTTTTTAG-3′
oligo (dT) 3	5′-CATTATGCTGAGTGATATCTTTTTTTTTCA-3′
oligo (dT) 4	5′-CATTATGCTGAGTGATATCTTTTTTTTTAA-3′
arbitrary 1	5′-ATTAACCCTCACTAAATCGGTCATAG-3′
arbitrary 2	5′-ATTAACCCTCACTAAATGGAGCTGG-3′
arbitrary 3	5′-ATTAACCCTCACTAAATGTGGCAGG-3′
arbitrary 4	5′-ATTAACCCTCACTAAAGCACCGTCC-3′
arbitrary 5	5′-ATTAACCCTCACTAAATGCTGGGGA-3′
arbitrary 6	5′-ATTAACCCTCACTAAATGCTGGTGG-3′
